# TNFR1 Absence Is Not Crucial for Different Types of Cell Reaction to TNF: A Study of the TNFR1-Knockout Cell Model

**DOI:** 10.3390/epigenomes8020015

**Published:** 2024-04-03

**Authors:** Alina A. Alshevskaya, Julia A. Lopatnikova, Julia V. Zhukova, Olga Y. Perik-Zavodskaia, Saleh Alrhmoun, Irina A. Obleukhova, Anna K. Matveeva, Darya A. Savenkova, Ilnaz R. Imatdinov, Dmitry V. Yudkin, Sergey V. Sennikov

**Affiliations:** 1 Federal State Autonomous Educational Institution of Higher Education I.M. Sechenov, First Moscow State Medical University of the Ministry of Health of the Russian Federation, 119435 Moscow, Russia; alkkina@yandex.ru (A.A.A.); lopatnikova18@yandex.ru (J.A.L.); zhukova1982@rambler.ru (J.V.Z.); saleh.alrhmoun1@gmail.com (S.A.); 2 Federal State Budgetary Scientific Institution, “Research Institute of Fundamental and Clinical Immunology” (RIFCI), 630099 Novosibirsk, Russiaobleukhova.irina@yandex.ru (I.A.O.); 3 Genome Research Department, State Research Center of Virology and Biotechnology “Vector”, Federal Service for Surveillance on Consumer Rights Protection and Human Well-Being (FBRI SRC VB “Vector”, Rospotrebnadzor), 630559 Koltsovo, Russia; annamatveeva516@gmail.com (A.K.M.); savenkova_da@vector.nsc.ru (D.A.S.); imatdinov_ir@vector.nsc.ru (I.R.I.); yudkin_dv@vector.nsc.ru (D.V.Y.)

**Keywords:** TNF receptors, TNFR1, TNFR2, gene knockout, flow cytometry, receptor expression density

## Abstract

Background: One of the mechanisms regulating the biological activity of tumor necrosis factor (TNF) in cells is the co-expression of TNFR1/TNFR2 receptors. A model with a differential level of receptor expression is required to evaluate the contribution of these mechanisms. Aim: The development of a cellular model to compare the effects of TNF on cells depending on the presence of both receptors and TNFR2 alone. Methods: TNFR1 absence modifications of ZR-75/1 and K-562 cell lines were obtained by TNFR1 knockout. The presence of deletions was confirmed by Sanger sequencing, and the absence of cell membrane receptor expression was confirmed by flow cytometry. The dose-dependent effect of TNF on intact and knockout cells was comparatively evaluated by the effect on the cell cycle, the type of cell death, and the profile of expressed genes. Results: Knockout of TNFR1 resulted in a redistribution of TNFR2 receptors with an increased proportion of TNFR2+ cells in both lines and a multidirectional change in the density of expression in the lines (increased in K562 and decreased in ZR75/1). The presence of a large number of cells with high TNFR2 density in the absence of TNFR1 in the K562 cells was associated with greater sensitivity to TNF-stimulating doses and increased proliferation but did not result in a significant change in cell death parameters. A twofold increase in TNFR2+ cell distribution in this cell line at a reduced expression density in ZR75/1 cells was associated with a change in sensitivity to low cytokine concentrations in terms of proliferation; an overall increase in cell death, most pronounced at standard stimulating concentrations; and increased expression of the lymphocyte-activation gene groups, host–pathogen interaction, and innate immunity. Conclusions: The absence of TNFR1 leads to different variants of compensatory redistribution of TNFR2 in cellular models, which affects the type of cell response and the threshold level of sensitivity. The directionality of cytokine action modulation and sensitivity to TNF levels depends not only on the fraction of cells expressing TNFR2 but also on the density of expression.

## 1. Introduction

Tumor necrosis factor alpha (TNF) is a multifunctional proinflammatory cytokine that plays an important role in many physiological and pathological processes [1]. The effects of TNF are not limited to the control of inflammatory responses; the cytokine can regulate a variety of cellular processes, from stimulation of cell proliferation and differentiation to activation of cell death programs such as apoptosis and necroptosis [2,3]. This wide variety of biological effects is made possible by the presence of two types of competing specific receptors: TNFR1 and TNFR2 (encoded by the TNFRSF1A and TNFRSF1B genes), which trigger different intracellular signaling pathways [4].

To date, a large number of mechanisms regulating the systemic and local effects of cytokines, and TNF in particular, have been identified [5]. These include not only cytokine serum levels and local expression of the membrane-bound form of cytokine but also various expression parameters and ratios of specific type 1 and type 2 receptors on cells and within subpopulations [6]. Currently, despite the large number of hypotheses, there is no unified understanding of what allows TNF to target the most important of its numerous biological effects [2,7].

Currently, one of the key hypotheses is the occurrence of differentiated interactions between different forms of cytokine and different forms of receptor complexes (soluble and membrane-bound forms) [8]. Thus, it is assumed that soluble forms of the cytokine are fully capable of activating only type 1 receptors (containing death domains), while the type 2 receptor is fully activated only through binding to the membrane-bound form of TNF [3]. However, there is no definite understanding of whether short-term binding of sTNF to type 2 receptors can lead to the activation of individual signaling pathways, or whether only cytokine transfer to type 1 receptors with the activation of pro-apoptotic pathways will occur [9,10]. A second hypothesis is the possibility of regulating the type and intensity of the interaction by changing the ligand–receptor ratio. At the same time, the influence of the cytokine level on the realized effects was proved both in experimental models [11] and in clinical medicine, while the influence of the number and the ratio of different types of receptors is currently being actively studied [6].

To answer these questions, it seems necessary to develop and study a model with various combinations of the presence of type 1 and type 2 receptors on the cell surface, which will allow us to evaluate the contribution of each type of receptor to the activation of certain signaling pathways and the possibility of isolated interaction of the cytokine with each of the receptors.

The aim of this study was to create cell lines with knockout of the TNFRSF1A gene to study the dose-dependent dynamics of cell response to TNF depending on the presence of one or two receptor types on their surface.

## 2. Materials and Methods

### 2.1. Cultivation of Cell Lines

Cell lines ZR-75/1 (breast carcinoma) and K-562 (chronic myelogenous leukemia) were selected for this work based on previously demonstrated type 1 and type 2 receptor co-expression profiles and differences in response to cytokine action [11]. Cell lines were provided by the Institute of Cytology, Russian Academy of Sciences (FSBI RAS, St. Petersburg, Russia).

Cells were thawed in a water bath at +37 °C for 1–2 min. Then, the cells were washed and cultured in an RPMI-1640 medium supplemented with 10% fetal bovine serum (FBS) (HyClone, Logan, UT, USA), 2 mM L-glutamine (BioloT LLC, Saint Petersburg, Russia), 5 × 10^−4^ M 2-mercaptoethanol (Sigma-Aldrich, Burlington, MA, USA), 80 μg/mL gentamicin (KRKA, Slovenia), 10 mM HEPES buffer (Sigma-Aldrich, USA), and 100 μg/mL benzylpenicillin (Biosintez, Penza, Russia) in an incubator in a humid atmosphere at 37 °C and CO_2_ concentration of 5%. After collection, centrifugation (for 10 min at 1500 rpm), and resuspension, the cells were transferred to plates (TPP, Switzerland) at the optimal density for each cell line. To evaluate the effect of TNFα on cell cultures, the cells were cultured for 72 h for K-562 and 96 h for ZR-75/1 in the absence and presence of recombinant human TNF (R&D Systems, Minneapolis, MN, USA) at various concentrations ranging from 0.01 to 5 ng/mL.

### 2.2. Plasmid Assembly for TNFRSF1A Gene Knockout

The vector expressing the *TNFRSF1A* gene knockout system was derived from the pSpCas9(BB)-2A-GFP vector (Addgene, #48138), as described previously [12].

Two single-guide RNAs (sgRNAs) were selected with CRISPOR version 4.99 (URL http://crispor.gi.ucsc.edu/ accessed on 20 November 2022), Benchling (URL https://www.benchling.com/ accessed on 20 November 2022), and CHOPCHOP version 3 (URL https://chopchop.cbu.uib.no/ accessed on 20 November 2022) open source software. Guides were predicted as the most effective and well-balanced based on both on-target and off-target characteristics. The oligonucleotides encoding sgRNAs 3.1 and 3.2—TNFRSF1A_G3.1_S_BbsI, TNFRSF1A_G3.1_AS_BbsI, TNFRSF1A_G3.2_S_BsaI and TNFRSF1A_G3.2_AS_BsaI (Table 1)—were hybridized to form Bso31I and BstV2I sticky ends and then cloned by Bso31I and BstV2I sites into pSpCas9(BB)-2A-GFP. *E. coli* NEB Stable (NEB) cells were transformed with the ligase mix. Colonies were screened for the presence of insertion by PCR using primer pairs 14R and TNFRSF1A_G3.1_S_BbsI for sgRNA3.1, and 14R and TNFRSF1A_G3.2_S_BsaI for sgRNA 3.2. The structure of the final plasmid was controlled by Sanger sequencing.

### 2.3. Transgenic Cell Line Production

Plasmid spCAS9_TNFRSF1A_G3.1_G3.2 was used for transfection to induce NHEJ after hydrolysis. A total of 1 μg of plasmid DNA was used to transfect 1.5 × 10^5^ cells. Transfection was performed by electroporation on a Neon™ Transfection System (Thermo Fisher Scientific, Waltham, MA, USA) according to the standard protocol in a 10 μL suspension volume and at 1350 V, with pulse width: 10 ms, and the number of pulses: 4. The cells were placed in a 24-well plate in 0.5 mL of support medium without antibiotics. Subcloning was performed using a Sony SH800 cell sorter in the FITC detection channel. At 12 h after transfection, single cells from each of the transfected cell cultures (K562 with spCAS9_TNFRSF1A_G3.1_G3.2; ZR-75-01 with spCAS9_TNFRSF1A_G3.1_G3.2) were sorted into the wells of a 96-well plate with 150 μL of the supporting medium, using 3 plates for each cell line: 2 plates were chosen for cells with a fluorescence intensity of 10^5^–10^6^ log RFU, and 1 plate was chosen for cells with a fluorescence intensity over 10^6^ log RFU. The deletion was screened by PCR with primers A-del_TNFRSF1A_3.2-F and A-del_TNFRSF1A_both_3.2+4.2-R (Table 1).

### 2.4. Flow Cytometry: Evaluation of TNF Receptor Expression and Co-Expression

**Figure 1:**
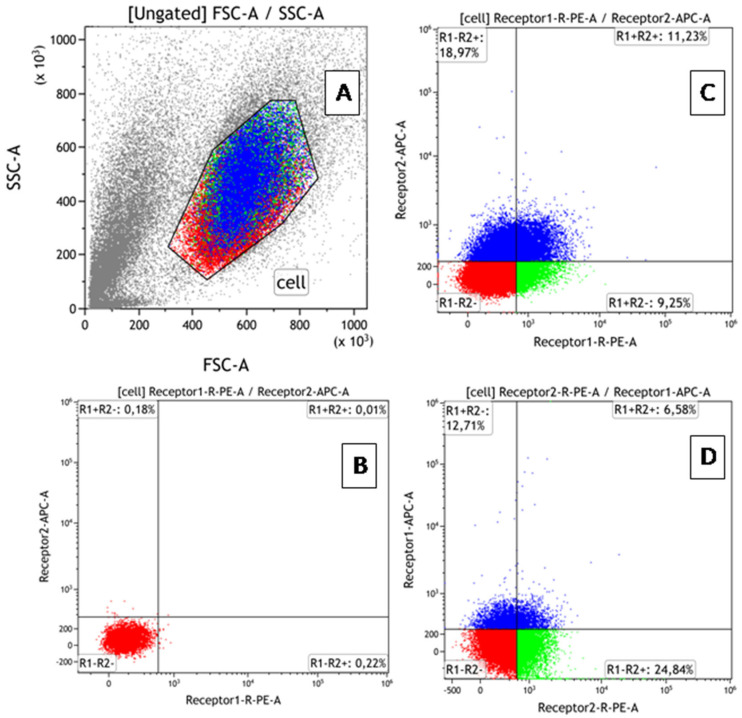
Flow cytometry gating protocol for identification of cells with TNFR1 and TNFR2 expression. (**A**) Forward scatter vs. side scatter plot used to identify mononuclear cells and lymphocytes. (**B**) Dot plot of PE/APC for samples without antibodies (control plot). (**C**) Use of antibodies TNFR1-PE + TNFR2-APC (experiment plot). (**D**) Use of antibodies TNFR1-APC + TNFR2-PE (experiment plot). Different colors show 4 fractions of cells co-expressing receptors in different combinations. The distribution of colors on a dot-plot with the phenotypic characteristics of the population shows that all cells are equivalent in size and density.

Phenotypic characteristics were assessed by flow cytometry (AttuneNxT cytofluorimeter (Thermo Fisher Scientific, Waltham, MA, USA)) using monoclonal antibodies: anti-humanTNFRI-RE, anti-humanTNFRII-RE, anti-humanTNFRI-APC, and anti-humanTNFRII-APC (R&D Systems, USA). Data processing and calculation of fluorescence intensity values were performed using the Attune™NxT software V3.2.1 (Thermo Fisher Scientific, Waltham, MA, USA). The BD QuantiBRITE PE kit (BD Biosciences, Franklin Lakes, NJ, USA) was used to create a calibration curve and translate fluorescence intensity values of cells expressing the corresponding marker into absolute values of receptor number. To prevent nonspecific binding, samples were incubated with an FcR inhibitor (BioLegend, San Diego, CA, USA) for 10 min at +4 °C, according to the manufacturer’s protocol, before staining the receptors. To simultaneously determine the percentage of co-expressing cells and count the number of receptors for TNF types 1 and 2 on cells in all 4 fractions of each cell line (TNFR1+TNFR2-, TNFR1+TNFR2+, TNFR1-TNFR2+, TNFR1-TNFR2-), double-labeled paired samples were performed. An example of gating is shown in Figure 1. Each sample with a specific dose of rhTNF (as well as a control sample without TNF) was divided into 2 tubes stained with TNFR1-PE + TNFR2-APC or TNFR1-APC + TNFR2-PE. After cytometric analysis, the number of type 1 receptors was calculated according to the BD QuantiBRITE PE protocol. The percentage of cells in each fraction was determined as the average between samples.

### 2.5. Flow Cytofluorimetry: Assessment of the Functional Activity of Cells

For cell cycle analysis, cells were collected after culturing and centrifuged in 1 mL PBS at 1500 rpm for 10 min followed by resuspension of the precipitate in 200 μL PBS with CytoPhase Violet commercial tag at a concentration of 0.5–1.0 × 10^6^ cells, according to the manufacturer’s protocol, and incubated for 90 min at +37 °C in a CO_2_ incubator, followed by the analysis on an AttuneNxT flow cytometer (Thermo Fisher Scientific, Waltham, MA, USA). When analyzed by anterior and lateral light scattering indices, the region corresponding to the cell population was distinguished. After the removal of doublets, a DNA histogram with the distribution of cell cycle phases was plotted on the CytoPhase Violet-A/CytoPhase Violet-H dot plot. The percentage of cells in each phase of the cell cycle was estimated.

To evaluate different phases of apoptosis in the selected cell lines, we used a commercial Annexin A5 apoptosis detection kit (BioLegend, San Diego, CA, USA). The cells were washed twice with cold BioLegend cell staining buffer and then resuspended in Annexin V binding buffer at a concentration of 0.25–1.0 × 10^7^ cells/mL. A sample of 5 μL of Annexin V FITC and 5 μL of 7-AAD viability staining solution were added and incubated for 15 min at room temperature (25 °C) in the dark, followed by analysis on an AttuneNxT flow cytometer (Thermo Fisher Scientific, Waltham, MA, USA). A region corresponding to the cell population was distinguished in the anterior (FSC) and lateral (SSC) light scattering analysis. A FITC Annexin versus 7-AAD dot plot was constructed from the total cell population gate, where Annexin+ 7AAD cells constituted the early apoptosis fraction. Then, from the Annexin+ 7AAD+ gate, we built a dot plot of FSC-H against FSC-A and chose the gate of cells with the highest intensity of luminescence. These cells made up the late apoptosis fraction from this gate. Next, we plotted a scatter plot of FSC versus 7AAD, where double-positive cells made up the necroptosis fraction.

### 2.6. Evaluation of the Profile of Expressed Genes in Cells upon Activation of Specific TNF Receptors

Total RNA was isolated from 250,000 sample cells using the Norgen Biotek Total RNA Purification Plus Kit according to the manufacturer’s instructions. The concentration and quality of RNA from each sample were assessed using a Nanodrop 2000 spectrophotometer (Thermo Fisher Scientific, Waltham, MA, USA). All samples were diluted to 15–20 ng/μL using nuclease-free water. Gene expression profiling using the Nanostring nCounter SPRINT Profiler assay system was performed on 100 ng of total RNA from each sample. RNA samples were analyzed using the nCounter Human Immunology v2 panel consisting of 579 immune- and inflammation-associated genes and 15 housekeeping genes as control samples. Samples were subjected to a hybridization reaction according to the manufacturer’s instructions, and after hybridization of the probes with the targets of interest in the samples, the number of target molecules was quantified using the nCounter digital analyzer and evaluated using the nSolver platform.

### 2.7. Statistical Analysis

Statistical analysis of the data was performed using the STATISTICA 7.0 software (StatSoft, Tulsa, OK, USA). To compare independent samples and calculate statistical significance, we used the Kruskal–Wallis test by ranks with multiple comparisons of median values (comparing the same indicators for different subpopulations and identifying differences between subgroups of subjects). To assess the dose-dependent effect of TNF on cell cycle parameters and to investigate associations between receptor expression and co-expression parameters and cell cycle parameters, correlation analysis was performed for each cell line culture using the Pearson correlation coefficient (at *p* < 0.05).

## 3. Results

### 3.1. TNFRSF1A Gene Deletion Characterization in Cells after Subcloning

**Figure 2:**
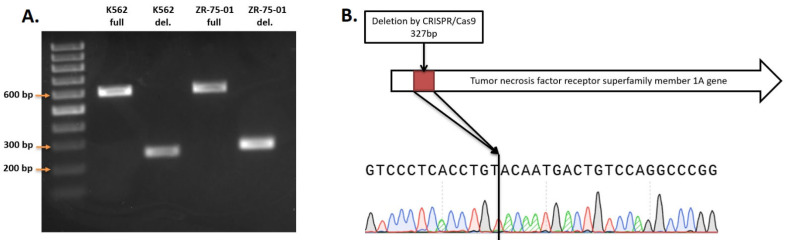
(**A**) PCR screening of *TNFRSF1A* gene deletion. (**B**) Sample of sequence of *TNFRSF1A* deletion in ZR-75-01 TNFRSF1A-\-9C9 cells.

We conducted knockout experiments aiming to generate homogeneous clonal cell lines, derived from K562 and ZR-75-01 cells, with biallelic *TNFRSF1A* gene knockout. A total of 9 clones with knockout in K562 cells and 21 clones from ZR-75-1 cells were obtained. The size of the fragment without deletion for PCR screening was 602 bp, and the size of the truncated fragment after deletion was about 275 bp (Figure 2A). The amplicons were sequenced by Sanger sequencing to determine the actual DNA sequence of the genes after the deletion (Figure 2B).

The *TNFRSF1A* gene in the generated cell lines contains a 327 bp deletion that leads to the absence of intron 2 and partial deletion of exons 2 and 3, forming a joint exon. Finally, the gene lost 102 bp of exonic sequences, leading to non-frame-shift deletion of the open reading frame. This mutation leads to the deletion of 34 amino acids in the extracellular domain of the protein, which should break its ligand interaction function.

### 3.2. Verification of Knockout Efficacy and Changes in TNFR2 Expression Levels in Knockout Lines

**Figure 3:**
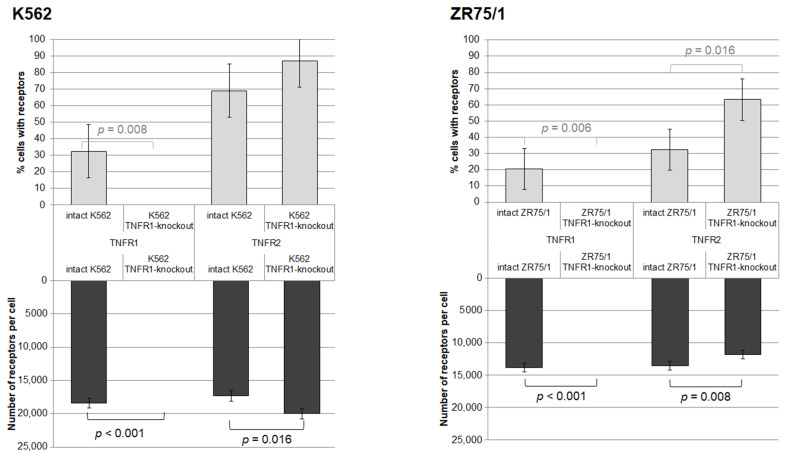
Effect of knockout of one of the receptors for TNF on the percentage of expressing cells and the density of receptor expression. %—percentage of cells expressing the corresponding receptor. N—the average number of expressed receptors on the cell surface. The number of replications, *n* = 5, for each of the experiments.

As a result of the series of cell line knockout experiments, two lines with TNFR1 knockout were successfully obtained. The efficacy of the knockout was confirmed by evaluating the level of type 1 and type 2 receptor expression in viable cells before and after transfection (Figure 3). Knockout resulted in the complete elimination of type 1 receptor detection in both lines. For the K562 line, there was a compensatory increase in the density of type 2 receptor expression in the cells (*p* = 0.031) with a trend toward an increased proportion of TNFR2+ cells (*p* = 0.055), whereas for the ZR75/1 line, there was a decrease in TNFR2 expression density (*p* = 0.008) with an increased proportion of TNFR2+ cells (*p* = 0.016).

### 3.3. Study of the Dose-Dependent Effect of Cytokine on the Functional Response of Cells Depending on Receptor Co-Expression

**Figure 4:**
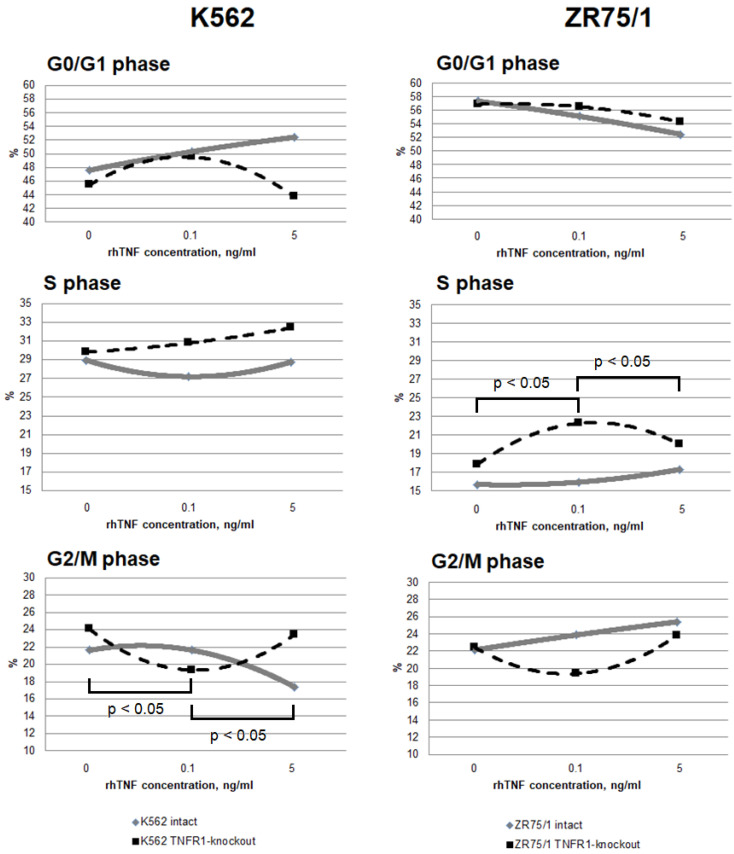
Dose-dependent effect of TNF cytokine on cell cycle parameters in intact and knockout cell lines. The cells were cultured for 72 h for K-562 and 96 h for ZR-75/1 in the absence and presence of recombinant human TNF at concentrations ranging from 0.01 to 5 ng/mL. The reagent CytoPhase Violet was added to the culture and incubated for 90 min at +37 °C. Analysis was performed using an AttuneNxT flow cytometer. Data are presented as the mean value. The number of replications is *n* ≥ 3 for each cell line.

**Figure 5:**
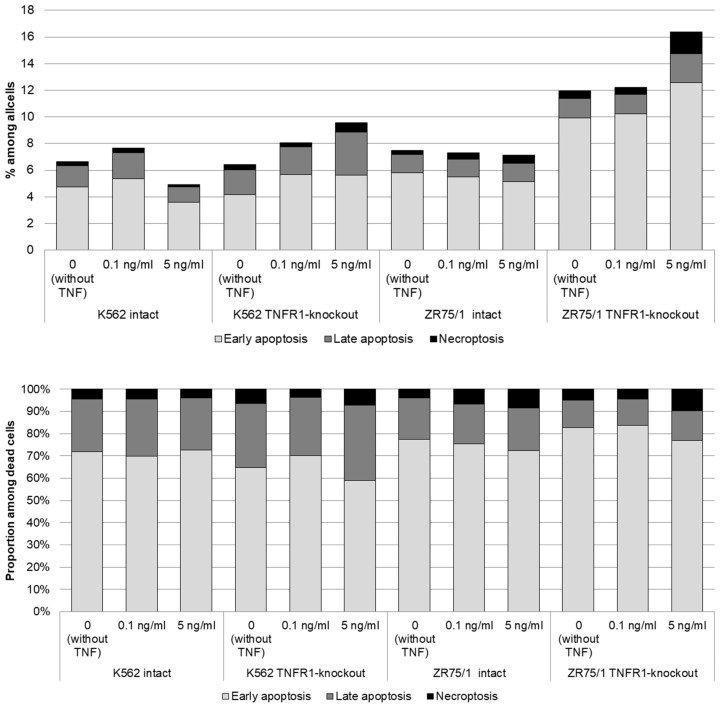
Dose-dependent effect of TNF cytokine on cell death parameters in intact and knockout cell lines. The cells were cultured for 72 h for K-562 and 96 h for ZR-75/1 in the absence and presence of recombinant human TNF at concentrations ranging from 0.01 to 5 ng/mL. The cells were stained using Annexin A5 apoptosis detection kit. Data are presented as the mean value. The number of replications is *n* ≥ 3 for each cell line.

To assess the contribution of co-expression and the presence of TNFR1 in the cells to the response to different cytokine concentrations, the dynamics of cell cycle phases (Figure 4) and types of cell death (Figure 5) were monitored.

It was shown that the addition of a minimum TNF-stimulating concentration of 0.1 ng/mL resulted in a significant redistribution of cells in the S and G2/M phases, with an increase in the proportion of the former and a decrease in the proportion of the latter in both TNFR1-knockout lines while maintaining the overall level of proliferation. A further increase in cytokine concentration led to a return of the parameters to the initial level. For the TNFR1-knockout lines, the minimum concentration was associated with a slight decrease in proliferation levels, while increasing the cytokine dose was associated with a decrease in proliferation levels and an increase in the proportion of cells in the G0/G1 phase.

More pronounced differences were obtained with respect to the proportion of cells at different stages of cell death. For both the K562 and ZR75/1 lines, no significant dose-dependent effect of TNF on the level of apoptosis was shown in the intact line. However, the knockout of the first type of receptor resulted in a significant increase in the level of apoptosis when the concentration of rhTNF increased. An important difference between the K562 and ZR75/1 knockout lines was the difference in the threshold level of cytokine response. For the intact and K562 knockout lines, a concentration of 0.1 ng/mL had an inhibitory effect, with an increase in the overall level of cell death. However, TNFR1 knockout for the ZR75/1 line resulted in a change in the cell response only to the standard stimulatory concentration of 5 ng/mL. For intact cells, this concentration was associated with an increase in proliferation and a significant decrease in apoptosis, whereas a significant increase in apoptosis was observed for the knockout line (*p* < 0.05).

To evaluate the dose-dependent changes in the functional activity of cells and to account for the influence of the receptor expression level on these parameters, a series of experiments were performed to simultaneously evaluate the cell cycle parameters and the receptor expression level when different concentrations of cytokine were added. Several significant differences and trends were identified. For the K562 line, it was found that although weak correlations were observed between receptor expression levels (in terms of the number of receptors expressed) and the cytokine dose, none of them were statistically significant (association between the TNF level and TNFR1 expression in the intact line: r = −0.279, *p* = 0.224; TNFR2 expression in the intact line: r = −0.268, *p* = 0.254; and TNFR2 expression in the TNFR1-knockout line: r = 0.103, *p* = 0.623). Only one trend was found between type 2 receptor expression density and cell death rate (TNFR2 density in TNFR1-knockout line and apoptosis rate, r = 0.541, *p* = 0.069). However, there were associations between TNFR2 expression density in the TNFR1-knockout K562 line and the overall proliferation level (r = 0.669, *p* = 0.0174) and the percentage of TNFR2+ cells in the same line and the proliferation level (r = 0.774, *p* = 0.031).

The evaluation of the effect of the cytokine level on the parameters of functional cell activity revealed some statistically significant associations. An increase in the cytokine dose was positively associated with an increase in proliferative activity in both intact lines (K562: r = 0.457, *p* = 0.0002; ZR75/1: r = 0.612, *p* = 0.0079) and in both TNFR1-knockout lines (K562: r = 0.595, *p* = 0.0005; ZR75/1: r = 0.649, *p* = 0.05). Regarding the effect on cell death, there was a trend toward a negative association between the cytokine dose and cell death in the TNFR1-knockout K562 line (r = −0.307, *p* = 0.099).

A limitation of this study is the analysis of apoptosis using population assessment of Annexin/7AAD staining. However, this approach has been successfully tested in a number of studies and shows a correlation with other methods [11,13].

### 3.4. Evaluation of the Profile of Expressed Genes in Cells upon Activation of Specific TNF Receptors

The ZR-75/1 line was chosen to further evaluate the effect of knockout on changes in functional cell activity, as it showed a multidirectional compensatory change in the proportion of positive cells and TNFR2 receptor expression density during TNFR1 knockout.

For the analysis, gene expression was examined using nCounter technology and the Human Immunology v2 panel to determine the transcriptional, regulatory, migration, differentiation, and metabolic mechanisms of immune cell interaction (Table 2). Analysis of differentially expressed genes in the ZR75/1 line showed increased expression of the following genes with increasing doses of TNF: ABCB1, ATG12, BCL6, BID, C1QA, C1S, CARD9, CCL24, CCL26, CD45RB, CD70, CD74, CD80, CD82, CIITA, EGR2, FCGR2B, GZMA, HFE, ICAM2, IL11RA, IL18RAP, IL1R1, IL23A, ITGAM, KLRG2, LAG3, MAP4K1, NCF4, NFKBIZ, NT5E, PIGR, PRF1, PRKCD, RAG1, TLR3, TNFS12, and TRAF6. Reduced gene expression with increased TNF concentration was shown for BCL3, C6, CCR10, CD4, CD55, CD96, CEACAM6, CR1, CSF3R, CXCL1, FCGR2A, FCGR2A/C, ICAM5, IFI35, IL16, IL18, IL1RAP, IL20, KLRC4, KLRF1, LTB4R, PTPN22, S1PR1, and SOCS3. A dose-dependent change in transcriptional activity was shown for each of the listed genes.

The majority of these genes belong to the cytokine signaling group, and the number of genes with increased and decreased expression is comparable. In the lymphocyte activation, host–pathogen interaction, and congenital immunity groups, the number of genes with increased expression, when TNF is added, is higher compared to the number of genes with suppressed expression. The chemokine signaling, TLR signaling, and complement system groups show a higher number of genes with increased expression, although the total number of DEGs in these groups is significantly lower. In the TNF family signaling, T-cell receptor signaling, and interferon type 1 signaling groups, the number of genes with suppressed activity is slightly higher than the number of genes with increased activity.

## 4. Discussion

It is now known that not only is the percentage of cells expressing specific receptors important for the formation of an effective response to a cytokine, but also the number of receptor molecules on the cell. The intensity and type of specific response formed may depend on the latter factor. In this case, it is necessary to take into account the parameter of simultaneous expression of both types of receptors on cells, since in this case it is the number of receptor molecules that will determine the final effect of the mediator on the cell. Determining the threshold level of receptor expression is an urgent task of modern molecular immunology.

Successful knockout of TNFR1 allowed us to obtain the modified cell lines ZR-75/1 and K-562 with knockout of one of the receptor types, the effectiveness of which was confirmed by flow cytometry data. A similar technique was used in various studies to evaluate different mechanisms of cytokine effect through the advanced receptor types. For example, plasmids with TNFR1 and TNFR2 gene knockout were used for the genetic modification of the cell line SK-Mel-37, and the efficiency of the knockout, as in this study, was evaluated by flow cytometry. The mean fluorescence intensity of the receptors in the knockout lines was determined at the level of the negative control [6]. This shows the efficacy of the receptor gene knockout-based technique for studying the mechanisms of cytokine and receptor interactions. In this study, we applied for the first time the technique of modifying two cell lines of different origins for further comprehensive evaluation of interaction mechanisms in the cytokine–receptor system, including dose-dependent changes in expression and co-expression, the cell cycle and apoptosis phases, and the cell transcriptome. This comprehensive approach expands the understanding of the interaction between TNF and its receptors.

Our previous studies used cell lines of various origins, in which it was shown that ZR75 and K562 have stable high expression of receptors for TNFR1 and TNFR2, while stimulation of these cell lines with various doses of TNF led to multidirectional changes in receptor expression, as well as changes in the functional response, manifested in the form of changes in apoptosis and the cell cycle [14]. Also, the determining factor was the production of stable clones with TNFR1 knockout specifically for these cell lines.

The Jurkat line was considered as a T-lymphocyte line, but data on receptor expression were not stable enough, the effect of the recombinant cytokine was not pronounced, and there were difficulties in obtaining an effective knockout. Therefore, this line was not used in this experiment.

Type 1- and type 2-specific receptor signaling variants are currently being actively studied and include at least five intracellular activation variants so far, leading to either cell survival, normal or atypical proliferation, or cell death [15,16,17]. This study aimed to compare the effects when two receptors and only one receptor are expressed simultaneously, which is an important regulatory mechanism. Recent studies established that the functional response of cells to cytokine action determined the formation of complexes I, IIa, IIb, and IIc. The formation of complex I leads to the activation of NF-κB, JNK, and p38 signaling, resulting in the production of proinflammatory molecules and triggering survival pathways. The formation of complexes IIa and IIb triggers apoptosis. The formation of complex IIc leads to the development of necroptosis [18]. It is also thought that activation of TNFR2 can trigger cell death; under certain conditions the receptor is able to activate a cell death process that is independent of TNFR1. Several studies indicate the existence of functional cross-reactions between TNFR1 and TNFR2, in which TNFR2 will act as an enhancer of the cytotoxic effect of TNFR1 [19].

Despite the large number of works aimed at studying the mechanisms of the interaction, the possible ways of targeting cytokine receptor regulation are currently unknown and represent a significant fundamental and applied scientific challenge [Sennikov 2019]. To test the existing hypotheses, a model is needed to identify the mechanisms of such regulation and assess the practical applicability of modulation of cytokine–receptor interaction to control the functional activity of target cells. The modeling method is widely used to study the mechanisms of TNF–receptor interaction. The models are cell lines of different origins, laboratory animals, and human blood cells. In animal studies, to study TNF-TNFR1/TNFR2 signaling, laboratory mice were injected with blocking antibodies, then stimulation was performed by injecting recombinant TNF, and activated genes of signaling pathways were evaluated [20]. The modulation of signaling pathways and the functional response of cells mediated by receptors were evaluated on human immunocompetent cells. The study evaluated TNFR2-specific signal transduction in Tfr and Treg cells, as well as the functional response of cells to the stimulation of this receptor [21]. Cell lines are also frequently used to evaluate signaling pathways and modulate the functional response. In a study of the effect of receptor conformational changes on the switching of the functional cell response, the HEK293 tumor cell line, which was knocked out by TNFR1, was used, followed by functional and kinetic tests [22]. The use of models in the present study provided new data on the mechanisms of regulation in the cytokine–receptor system, and the development and validation of a model of cell lines of the same origin with different combinations of TNF receptor expression provide a practical tool for solving such problems.

Blocking the TNF/TNFR pathway can be performed at various levels, resulting in different functional cell responses. In particular, blocking TNFR1 on the cell surface leads to changes in the functional response of cells. It was shown that the use of etarnecept, which is a competitive inhibitor of the binding of TNF to its receptors, on the endothelial cell line EA.hy926 did not lead to significant changes in apoptosis, while the combined use of etarnecept and TNF caused a decrease in TNF-induced apoptosis [23]. Similarly, in our study, gene deletion led to damage to the extracellular domain of the protein, which occurred in the absence of ligand–receptor binding, and we observed changes in the functional response of cells in response to stimulation.

A comparison of the data on the effect of knockout of one receptor on receptors of another type allowed us to establish that complete inactivation of the expression of receptors of the first type leads to a compensatory increase in the expression of receptors of the second type due to an increase in the proportion of expressing cells. At the same time, differently directed knockout effects on the expression density of the other receptor type were established.

We hypothesize that this difference may be due not only to the different origins of the lines, but also to the initially significantly lower expression of type 2 receptors on ZR75/1 (both in % and in density) compared to K562. Studies showed that TNFR1 expression on monocytes was decreased when TNFR2 was selectively blocked, and conversely, TNFR2 activation resulted in increased TNFR1 expression [24]. Molecular mechanisms of interaction between the two types of receptors were also shown. Co-expression of TNFR1 with mutant TNFR2 receptors unable to bind or degrade TRAF2 enhances the role of the adaptor protein, with NF-kB activation occurring only when TNFR2 cannot induce TRAF2 depletion. Conversely, the cytotoxic activity of TNFR1 increases when the receptor is expressed together with the TNFR2 receptor, which induces TRAF2 degradation [25]. The obtained data confirm the mutual influence of the two receptor types and reveal the peculiarities of receptor expression changes depending on the type of cell line and the initial level of expression.

In this study, we assessed the functional activity of cells under the dose-dependent influence of the cytokine TNF. The revealed associations and trends demonstrated that although certain cytokine concentrations were associated with the initiation of signaling pathways prioritized for survival or cell death, the intensity of these cellular effects could be regulated by receptor expression density indices and receptor co-expression parameters. A dose-dependent effect of TNF on receptor expression was shown for other cell lines. In a previous study, IPEC-J2 cells were incubated with TNF for 48 h at concentrations of 500, 1000, and 5000 U/mL and showed a dose-dependent increase in TNFR1 expression, while TNFR2 expression remained the same [26]. It is also believed that low concentrations of TNF-α tend to stimulate cell proliferation, whereas high concentrations tend to inhibit cell proliferation and even induce apoptosis, with biological effects increasing in relation to TNF concentration [27]. This work allows us to determine the parameters of cell response to different doses of cytokine.

Differences in differentially expressed genes in terms of the prevalence of genes with increased expression were most pronounced in the groups of lymphocyte activation, host–pathogen interaction, and innate immunity and less pronounced in the groups of chemokine signaling, TLR signaling, and the complement system. The findings confirmed the presence of significant changes in gene expression with changes in the TNF dose. Changes in gene expression in response to TNF exposure were shown in other studies. Incubation with TNF causes dramatic changes in transcription programs in promyelocytic and granulocytic HL-60/S4 cells, inducing a canonical TNF response involving NKFB, p53, inflammatory signaling, and apoptosis [28].

There are studies showing that TNF stimulation leads to changes in the transcriptome of mononuclear cells [29]. However, our study provides previously unexplored data showing the effect of different doses of TNF on receptor expression and changes in the proliferation, apoptosis, and transcriptome of tumor cell lines of various origins. The data obtained require additional research, but they have great potential for fundamental and clinical application.

## 5. Conclusions

Evaluation of the type and nature of the response of tumor cell lines of different genesis to different doses of cytokine showed that the cellular response to TNF depended on the expression density of TNFR1 and TNRF2 as well as on their co-expression on cells. Modified variants of lines of different origin (ZR-75/1 and K-562) co-expressing both types of TNF receptor, with TNFR1 gene knockout, were obtained. A comparison of the modified lines of different origin in terms of functional response to the cytokine allowed us to establish the possibility of modulating the action of the cytokine by changing the level of receptor expression in the cells.

## Figures and Tables

**Table 1:** Oligonucleotides used in cell production.

Name	Sequence 5′-3′	Reference
TNFRSF1A_G3.1_S_BbsI	CACCGGTGGACTGGTCCCTCACCTAG	GenBank: AH003016.2
TNFRSF1A_G3.1_AS_BbsI	AAACCTAGGTGAGGGACCAGTCCACC	GenBank: AH003016.2
TNFRSF1A_G3.2_S_BsaI	TGCTGGTGGACAGTCATTGTACAAGT	GenBank: AH003016.2
TNFRSF1A_G3.2_AS_BsaI	AAACACTTGTACAATGACTGTCCACC	GenBank: AH003016.2
14R	ATAAGGTCATGTACTGGGCACA	Addgene, #48138
A-del_TNFRSF1A_3.2-F	CCACACACCACTCAAGACC	GenBank: AH003016.2
A-del_TNFRSF1A_both_3.2+4.2-R	CCCTCCCTCTCTTGATGGTGTC	GenBank: AH003016.2

**Table 2:** Summary table of changes in the transcriptome profile in ZR75/1 line samples upon addition and upregulation of TNF. Genes with increased expression are marked in green, and genes with decreased expression are marked in red.

Gene	Cytokine Signaling	Lymphocyte Activation	Host-Pathogen Interaction	Innate Immune System	Adaptive Immune System	Immunometabolism	NLR Signaling	Oxidative Stress	Chemokine Signaling	Complement System	Hemostasis	TLR Signaling	TNF Family Signaling	Apoptosis	Th17 Differentiation	Phagocytosis and Degradation	Cell Adhesion	Transcriptional Regulation	Autophagy	T Cell Receptor Signaling	Type II Interferon Signaling	Lymphocyte Trafficking	MHC Class I Antigen Presentation	MHC Class II Antigen Presentation	NF-kB Signaling	Type I Interferon Signaling	B Cell Receptor Signaling	Inflammasomes
PRKCD	+	+	−	+	−	−	+	+	+	−	+	−	−	+	−	−	−	−	+	−	+	−	−	−	−	−	−	−
TRAF6	+	+	+	+	+	−	+	−	−	−	−	+	−	−	−	−	−	−	+	+	−	−	−	−	+	−	−	−
ITGAM	+	−	+	+	−	−	−	−	−	+	+	+	−	−	−	+	+	−	−	−	−	+	−	−	−	−	−	−
SOCS3	+	−	+	−	+	−	−	−	−	−	−	−	+	−	−	−	−	−	−	−	+	−	+	−	−	+	−	−
APP	+	−	−	+	−	−	+	+	−	−	+	+	−	−	−	−	−	−	−	−	−	−	−	−	−	−	−	+
CD4	+	+	−	+	+	−	−	−	−	−	−	−	−	−	−	−	+	−	−	+	−	−	−	−	−	−	−	−
CXCL1	+	−	+	+	−	−	+	−	+	−	−	−	+	−	−	−	−	−	−	−	−	−	−	−	−	−	−	−
NCF4	−	−	+	+	+	−	−	−	−	−	−	−	−	−	−	+	−	−	−	−	−	+	+	−	−	−	−	−
CXCL10	+	−	+	−	−	−	−	−	+	−	−	+	+	−	−	−	−	−	−	−	−	−	−	−	−	−	−	−
ABL1	−	+	+	+	−	−	−	+	−	−	+	−	−	−	−	−	−	−	−	−	−	−	−	−	−	−	−	−
CD74	−	+	+	−	+	−	−	−	−	−	+	−	−	−	−	−	−	−	−	−	−	−	−	+	−	−	−	−
CD80	+	+	−	−	+	−	−	−	−	−	−	+	−	−	−	−	+	−	−	−	−	−	−	−	−	−	−	−
IL1R1	+	−	+	−	−	−	−	+	−	−	−	−	−	−	+	−	−	−	−	−	−	−	−	−	+	−	−	−
IL18	+	+	+	−	−	−	+	−	−	−	−	−	−	−	−	−	−	−	−	−	−	−	−	−	−	−	−	−
AHR	−	+	−	−	−	+	−	−	−	−	−	−	−	−	+	−	−	+	−	−	−	−	−	−	−	−	−	−
ARG1	−	−	+	+	−	+	−	+	−	−	−	−	−	−	−	−	−	−	−	−	−	−	−	−	−	−	−	−
FCGR2B	−	−	+	−	+	−	−	−	−	−	−	−	−	−	−	+	−	−	−	−	−	−	−	−	−	−	+	−
ICAM2	−	+	−	+	+	−	−	−	−	−	−	−	−	−	−	−	+	−	−	−	−	−	−	−	−	−	−	−
IL23A	+	+	+	−	−	−	−	−	−	−	−	−	−	−	+	−	−	−	−	−	−	−	−	−	−	−	−	−
BCL3	−	+	−	−	−	−	−	−	−	−	−	−	+	−	−	−	−	+	−	−	−	−	−	−	−	−	−	−
CD55	−	+	−	+	−	−	−	−	−	+	−	−	−	−	−	−	−	−	−	−	−	−	−	−	−	−	−	−
CR1	−	−	+	+	−	−	−	−	−	+	−	−	−	−	−	−	−	−	−	−	−	−	−	−	−	−	−	−
PTPN22	−	+	−	−	+	−	−	−	−	−	−	−	−	−	−	−	−	−	−	+	−	−	−	−	−	−	−	−
ADA	−	+	−	−	−	+	−	+	−	−	−	−	−	−	−	−	−	−	−	−	−	−	−	−	−	−	−	−
ATG12	−	−	−	+	−	−	+	−	−	−	−	−	−	−	−	−	−	−	+	−	−	−	−	−	−	−	−	−
BID	−	+	+	−	−	−	−	−	−	−	−	−	−	+	−	−	−	−	−	−	−	−	−	−	−	−	−	−
C1QA	−	−	+	+	−	−	−	−	−	+	−	−	−	−	−	−	−	−	−	−	−	−	−	−	−	−	−	−
C1S	−	−	+	+	−	−	−	−	−	+	−	−	−	−	−	−	−	−	−	−	−	−	−	−	−	−	−	−
CARD9	−	−	+	+	−	−	+	−	−	−	−	−	−	−	−	−	−	−	−	−	−	−	−	−	−	−	−	−
CIITA	+	−	+	−	−	−	−	−	−	−	−	−	−	−	−	−	−	−	−	−	+	−	−	−	−	−	−	−
LAG3	−	+	−	−	+	−	−	−	−	−	−	−	−	−	−	−	−	−	−	−	−	−	−	+	−	−	−	−
TLR3	−	−	+	+	−	−	−	−	−	−	−	+	−	−	−	−	−	−	−	−	−	−	−	−	−	−	−	−
C6	−	−	−	+	−	−	−	−	−	+	−	−	−	−	−	−	−	−	−	−	−	−	−	−	−	−	−	−
CCR10	+	−	−	−	−	−	−	−	+	−	−	−	−	−	−	−	−	−	−	−	−	−	−	−	−	−	−	−
CEACAM6	−	−	−	+	−	−	−	−	−	−	+	−	−	−	−	−	−	−	−	−	−	−	−	−	−	−	−	−
FCGR2A/C	−	−	+	−	−	−	−	−	−	−	−	−	−	−	−	+	−	−	−	−	−	−	−	−	−	−	−	−
IFI35	+	−	−	−	−	−	−	−	−	−	−	−	−	−	−	−	−	−	−	−	−	−	−	−	−	+	−	−
IL1RAP	+	−	−	−	−	−	−	−	−	−	−	−	−	−	+	−	−	−	−	−	−	−	−	−	−	−	−	−
ARG2	−	−	+	−	−	+	−	−	−	−	−	−	−	−	−	−	−	−	−	−	−	−	−	−	−	−	−	−
BCL6	+	+	−	−	−	−	−	−	−	−	−	−	−	−	−	−	−	−	−	−	−	−	−	−	−	−	−	−
CCL24	+	−	−	−	−	−	−	−	+	−	−	−	−	−	−	−	−	−	−	−	−	−	−	−	−	−	−	−
CCL26	+	−	−	−	−	−	−	−	+	−	−	−	−	−	−	−	−	−	−	−	−	−	−	−	−	−	−	−
CD70	+	+	−	−	−	−	−	−	−	−	−	−	−	−	−	−	−	−	−	−	−	−	−	−	−	−	−	−
EGR2	−	−	+	−	−	−	−	−	−	−	−	−	−	−	−	−	−	+	−	−	−	−	−	−	−	−	−	−
GZMA	−	+	−	−	−	−	−	−	−	−	−	−	−	+	−	−	−	−	−	−	−	−	−	−	−	−	−	−
IL18RAP	+	−	−	−	−	−	−	+	−	−	−	−	−	−	−	−	−	−	−	−	−	−	−	−	−	−	−	−
IL1R2	+	−	+	−	−	−	−	−	−	−	−	−	−	−	−	−	−	−	−	−	−	−	−	−	−	−	−	−
IL1RAP	+	−	−	−	−	−	−	−	−	−	−	−	−	−	+	−	−	−	−	−	−	−	−	−	−	−	−	−
PRF1	−	+	−	−	−	−	−	−	−	−	−	−	−	+	−	−	−	−	−	−	−	−	−	−	−	−	−	−
RAG1	+	+	−	−	−	−	−	−	−	−	−	−	−	−	−	−	−	−	−	−	−	−	−	−	−	−	−	−
CD96	−	−	−	−	+	−	−	−	−	−	−	−	−	−	−	−	−	−	−	−	−	−	−	−	−	−	−	−
CSF3R	+	−	−	−	−	−	−	−	−	−	−	−	−	−	−	−	−	−	−	−	−	−	−	−	−	−	−	−
ICAM5	−	−	−	−	+	−	−	−	−	−	−	−	−	−	−	−	−	−	−	−	−	−	−	−	−	−	−	−
IL16	+	−	−	−	−	−	−	−	−	−	−	−	−	−	−	−	−	−	−	−	−	−	−	−	−	−	−	−
IL20	+	−	−	−	−	−	−	−	−	−	−	−	−	−	−	−	−	−	−	−	−	−	−	−	−	−	−	−
KLRC4	−	+	−	−	−	−	−	−	−	−	−	−	−	−	−	−	−	−	−	−	−	−	−	−	−	−	−	−
KLRF1	−	−	−	−	+	−	−	−	−	−	−	−	−	−	−	−	−	−	−	−	−	−	−	−	−	−	−	−
LTB4R	−	−	−	−	−	+	−	−	−	−	−	−	−	−	−	−	−	−	−	−	−	−	−	−	−	−	−	−
S1PR1	+	−	−	−	−	−	−	−	−	−	−	−	−	−	−	−	−	−	−	−	−	−	−	−	−	−	−	−
ABCB1	−	−	−	−	−	+	−	−	−	−	−	−	−	−	−	−	−	−	−	−	−	−	−	−	−	−	−	−
AICDA	−	+	−	−	−	−	−	−	−	−	−	−	−	−	−	−	−	−	−	−	−	−	−	−	−	−	−	−
AIRE	−	+	−	−	−	−	−	−	−	−	−	−	−	−	−	−	−	−	−	−	−	−	−	−	−	−	−	−
CD82	−	−	−	−	−	−	−	−	−	−	−	−	−	+	−	−	−	−	−	−	−	−	−	−	−	−	−	−
HFE	−	+	−	−	−	−	−	−	−	−	−	−	−	−	−	−	−	−	−	−	−	−	−	−	−	−	−	−
IL11RA	+	−	−	−	−	−	−	−	−	−	−	−	−	−	−	−	−	−	−	−	−	−	−	−	−	−	−	−
KLRG2	−	+	−	−	−	−	−	−	−	−	−	−	−	−	−	−	−	−	−	−	−	−	−	−	−	−	−	−
MAP4K1	−	−	−	−	−	−	−	−	−	−	−	−	+	−	−	−	−	−	−	−	−	−	−	−	−	−	−	−
NFKBIZ	−	−	−	−	−	−	−	−	−	−	−	−	−	−	−	−	−	+	−	−	−	−	−	−	−	−	−	−
NT5E	−	−	−	−	−	+	−	−	−	−	−	−	−	−	−	−	−	−	−	−	−	−	−	−	−	−	−	−
PIGR	−	−	−	+	−	−	−	−	−	−	−	−	−	−	−	−	−	−	−	−	−	−	−	−	−	−	−	−

## Data Availability

The data presented in this study are available on request from the corresponding author.
